# Risk factors in the development of gastric adenocarcinoma in the general population: A cross-sectional study of the Wuwei Cohort

**DOI:** 10.3389/fmicb.2022.1024155

**Published:** 2023-01-12

**Authors:** Zhaofeng Chen, Ya Zheng, Ping Fan, Min Li, Wei Liu, Hao Yuan, Xin Liu, Zhiyi Zhang, Zhengqi Wu, Yuping Wang, Rui Ji, Qinghong Guo, Yuwei Ye, Jinhua Zhang, Xiaohua Li, Feng An, Linzhi Lu, Youpeng Li, Xiang Wang, Jun Zhang, Quanlin Guan, Qiang Li, Min Liu, Qian Ren, Xiaobin Hu, Hong Lu, Hongling Zhang, Yue Zhao, Xi Gou, Xiaochuang Shu, Jun Wang, Zenan Hu, Siqian Xue, Jiankang Liu, Yongning Zhou

**Affiliations:** ^1^Department of Gastroenterology, The First Hospital of Lanzhou University, Lanzhou, Gansu, China; ^2^Key Laboratory for Gastrointestinal Diseases of Gansu Province, The First Hospital of Lanzhou University, Lanzhou, Gansu, China; ^3^Gansu Wuwei Tumor Hospital, Wuwei, Gansu, China; ^4^School of Basic Medical Sciences, Lanzhou University, Lanzhou, Gansu, China; ^5^Department of Gastroenterology, The 940th Hospital of Joint Service Logistics Support Force of Chinese People's Liberation Army, Lanzhou, Gansu, China; ^6^Gansu Second Provincial People’s Hospital, Lanzhou, Gansu, China; ^7^Wuwei Liangzhou Hospital, Wuwei, Gansu, China; ^8^Wuwei People's Hospital, Wuwei, Gansu, China; ^9^Minqin People's Hospital, Minqin, Gansu, China; ^10^Lanzhou University Second Hospital, Lanzhou, Gansu, China; ^11^Surgical Oncology Department, The First Hospital of Lanzhou University, Lanzhou, Gansu, China; ^12^School of Public Health, Lanzhou University, Lanzhou, Gansu, China; ^13^Interventional Radiology Department, The First Hospital of Lanzhou University, Lanzhou, Gansu, China; ^14^Harvard Medical School, Cardiovascular Division, Brigham and Women’s Hospital, Boston, MA, United States

**Keywords:** *Helicobacter pylori*, gastric adenocarcinoma, premalignant lesions, risk factors, progression

## Abstract

Several risk factors have been identified for the development of gastric adenocarcinoma (GAC), where the control group was usually a healthy population. However, it is unclear at what stage known risk factor exert their influence toward the progression to cancer. Based on the Wuwei Cohort, we enrolled 1,739 patients with chronic non-atrophic gastritis (no-CAG), 3,409 patients with chronic atrophic gastritis (CAG), 1,757 patients with intestinal metaplasia (IM), 2,239 patients with low-grade dysplasia (LGD), and 182 patients with high-grade dysplasia (HGD) or GAC to assess the risk factors between each two consecutive stages from no-CAG to GAC/HGD using adjusted logistic regression. We found that different groups of risk factors were associated with different stages. Age, occupation of farmer, low annual family income, *Helicobacter pylori* (*H. pylori*) infection, drinking, eating hot food, histories of gastritis and peptic ulcer were associated with the development of CAG. Age, illiteracy, *H. pylori* infection, smoking, eating hot food, eating quickly, and histories of gastritis and gallbladder diseases were associated with the progression to IM from CAG. Male, occupation of farmer and history of peptic ulcer were associated with the development of LGD from IM. Age, male and polyp history appeared to be risk factors associated with the development of GAC/HGD from LGD. In conclusion, it seems that most risk factors function more as a set of switches that initiated the GAC carcinogenesis. *H. Pylori* eradication and control of other risk factors should be conducted before IM to decrease the incidence of GAC.

## 1. Introduction

Gastric adenocarcinoma (GAC) is the most common histological type of gastric cancer (~95%). In 2018, GAC was diagnosed in approximately 1,033,701 individuals, leading to 782,685 deaths worldwide, making it the second leading cause of cancer-related deaths (Bray et al., 2018). Several risk factors have been identified for GAC, including age, male sex, low socioeconomic status, *Helicobacter pylori* (*H. pylori*) infection, smoking, drinking, and the presence of precancerous conditions. As each risk factor is common, it is difficult to identify high-risk populations of GAC (Meng et al., 2015; Yoon and Kim, 2015; Berger et al., 2016; Van Cutsem et al., 2016).

Most GAC follows a stepwise progression from normal gastric mucosa to chronic non-atrophic gastritis (no-CAG), chronic atrophic gastritis (CAG), intestinal metaplasia (IM), low-grade dysplasia (LGD), high-grade dysplasia (HGD), and GAC (e.g., Correa’s Cascade; Correa, 1992; Correa and Piazuelo, 2012). Risk factors that have been identified for the overall progression to cancer, where the healthy population was always used as the reference group, may pose a risk for an intermediary stage, but possibly less so for the final transition from dysplasia to cancer. It is unclear to what extent known risk factors are responsible for the development of different disease stages from normal gastric mucosa to cancer.

In this study, we included patients pathologically diagnosed with normal gastric mucosa, no-CAG, CAG, IM, dysplasia, and GAC from the baseline data of the Wuwei Cohort. We assessed the risk factors for different disease stages on the path to GAC. This study aimed to examine which group of known risk factors to what extent influence different disease stages of Correa’s Cascade.

## 2. Materials and methods

### 2.1. Study population

**Figure 1:**
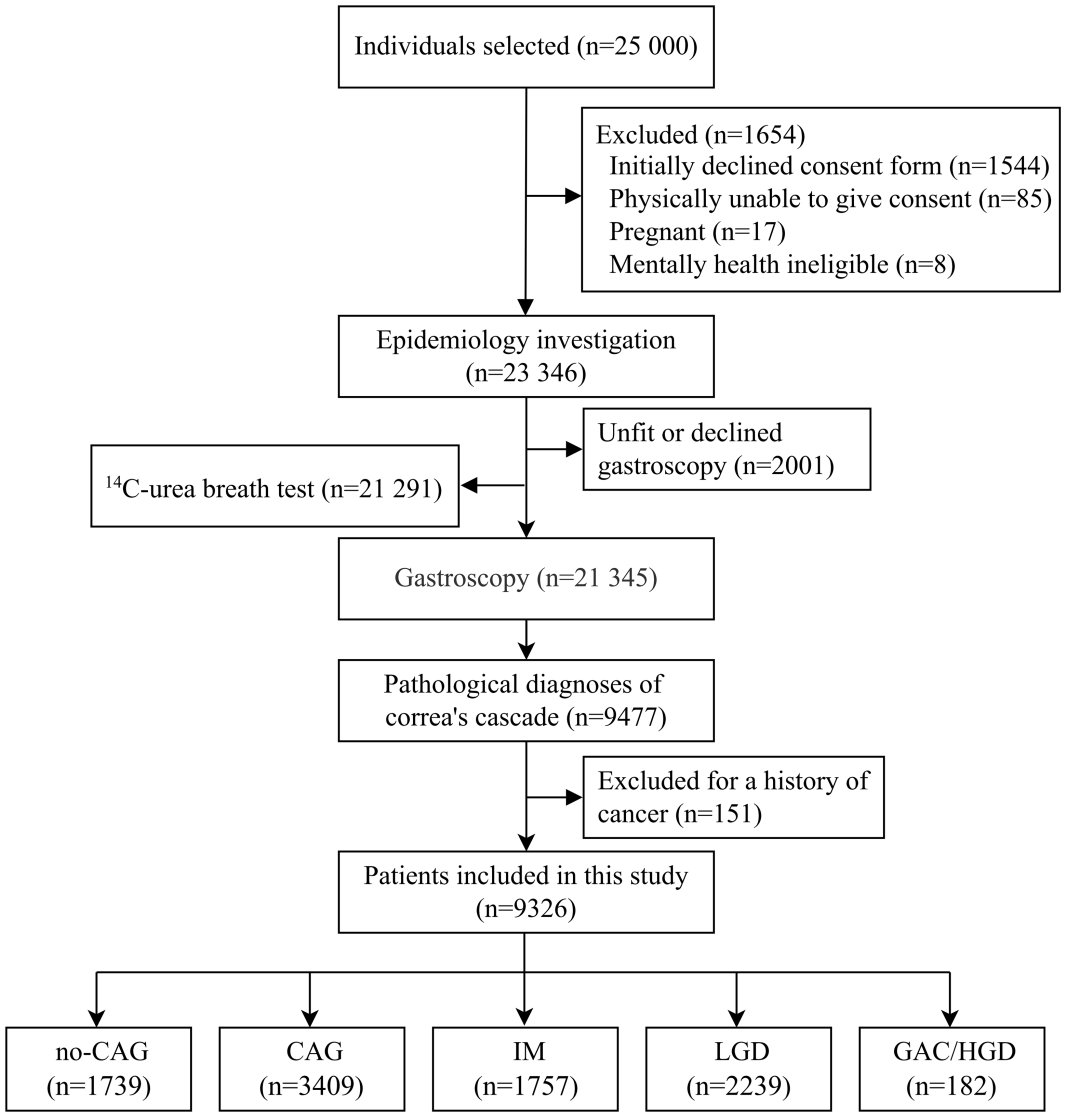
Flow chart of the study population. This figure depicts the study population screening process and the inclusion and exclusion criteria. no-CAG, chronic non-atrophic gastritis; CAG, chronic atrophic gastritis; IM, intestinal metaplasia; LGD, low-grade dysplasia; HGD, high-grade dysplasia; GAC, gastric adenocarcinoma.

Detailed descriptions of the Wuwei Cohort study design and characteristics of the participants have been provided elsewhere (Ji et al., 2021). In brief, the Wuwei Cohort is a population-based cohort of gastric cancer in Wuwei Municipality where the incidence and mortality rates of GAC are among the highest in China (Chen et al., 2016; Li et al., 2016; Liu et al., 2016). A total of 23,346 eligible participants aged 35–70 years comprised the Wuwei Cohort. Of these 23,346 participants, 21,345 and 21,291 participants underwent gastroscopic examination and *H. pylori* detection, respectively. Of the 21,345 participants who underwent gastroscopic examinations, 9,477 had gastric pathological diagnoses of normal gastric mucosa, no-CAG, CAG, IM, LGD, HGD, and GAC. In the present study, 9,326 participants were included after excluding participants with a history of cancer (Figure 1). Because no-CAG is very common in people aged >40 years and typically not considered at increased risk for gastric cancer, participants diagnosed with no-CAG and normal gastric mucosa were grouped as the no-CAG group in this study. In addition, participants with HGD and intestinal-type GAC were grouped as the GAC/HGD group, because HGD may share the same risk factors as cancer (Pohl et al., 2013). The Ethics Committee of The First Hospital of Lanzhou University approved the study (approval number: LDYYLL2012001) and written informed consent was obtained from all participants according to the Declaration of Helsinki.

### 2.2. Exposure and outcome assessment

In this study, covariates included sociodemographic characteristics (age, sex, education, occupation, marital status, and household income), smoking, drinking, diet habits (eating hot food and eating quickly), body mass index (BMI), *H. pylori* infection status, and medical history (gastritis, peptic ulcer, hepatitis, pancreatitis, gallbladder diseases, polyp, high blood pressure, diabetes, and anemia). Participants were classified as smokers if they had smoked at least one cigarette per day in the past 6 months or ever smoked, while they were defined as alcohol consumers if they consumed at least 1,000 g of beer, 150 g of wine, or hard liquor at least once per week during the past 1 year. Participants were defined as eating hot food if they reported a habit of eating hot food. The habit of eating quickly was defined if participants finished a bowl of noodles in 8 min or less. Height and weight were measured by trained staff, and BMI was defined as the weight (in kilograms) divided by the square of height (in meters). Then, BMI was categorized according to the Chinese cut-points: BMI < 18.5 kg/m^2^ for underweight, 18.5 kg/m^2^ ≤ BMI < 24 kg/m^2^ for normal-weight, 24 kg/m^2^ ≤ BMI < 28.0 kg/m^2^ for overweight, and BMI ≥ 28.0 kg/m^2^ for obesity. We used ^14^C-Urea breath test to determine the active *H. pylori* infection. The participants swallowed a test capsule that contains urea tagged with radioactive carbon 14 with water on an empty stomach or 2 h after eating. The ^14^C labeled urea was detected using an *H. pylori* detector (Shenzhen Zhonghe Headway BIO-SCI & TECH, China). Participants were considered to have hypertension if they were taking antihypertensive medications, self-reported a prior diagnosis of hypertension, and/or if their systolic pressure was ≥140 mmHg or diastolic pressure was ≥90 mmHg at baseline. Diabetes was defined as a fasting blood glucose level ≥ 7.0 mmol/L, or a self-reported prior diagnosis of diabetes at baseline. Gastroscopic examinations were performed by trained physicians using conventional white light GIFH 260 or 290 gastroscopy (Olympus, Japan), and biopsy specimens were obtained from the gastric body, angulus, and antrum according to the study protocol adapted from the Updated Sydney System. Histological biopsy specimens were examined by a panel of experienced pathologists based on the WHO classification (Fred et al., 2010). Participants with multiple lesions were categorized according to the severity of the lesion.

### 2.3. Statistical analysis

Comparison of means, medians, or frequencies between two consecutive disease stages (no-CAG and CAG, CAG and IM, IM and LGD, and LGD and GAC/HGD) were performed using Student’s *t*-test, Mann–Whitney *U* test, χ^2^ test, or Fisher’s exact probability test where appropriate. Multivariable binary logistic regression was used to calculate adjusted odds ratios (ORs) with 95% confidence intervals (CIs) between two consecutive disease stages. The variables included in the adjusted model were determined based on the comparison between the GAC/HGD and no-CAG groups using a stepwise multinomial logistic regression. *p*-values < 0.05 are considered significant. All statistical analyses of data were performed using Stata 15.0 (StataCorp LLC, College Station, TX, United States).

## 3. Results

### 3.1. Patient characteristics

A total of 9,326 participants were included in the study. Of these, 1,739 participants were included in the no-CAG group (222 participants with normal gastric mucosa and 1,517 with no-CAG), 3,409 participants in the CAG group, 1,757 in the IM group, 2,239 in the LGD group, and 182 in the GAC/HGD group (74 participants with HGD and 108 participants with intestinal-type GAC).

Table 1 shows the detailed distribution of the factors between the disease stages. Compared with the no-CAG group, patients with CAG were older, more likely to be farmers and low-income earners, more likely infected with *H. pylori*, more likely to consume alcohol and eat hot food, and had histories of gastritis and peptic ulcer. Compared to patients with CAG, patients with IM were older, more likely to be uneducated, to be farmers, smokers, hot food eaters, fast eaters, and had histories of gastritis and gallbladder disease. Age was not significantly different between patients with IM and those with LGD. Patients with LGD were more often men, more likely to be smokers, less likely to eat hot food, and had a history of peptic ulcer and hepatitis compared to patients with IM. Compared to patients with LGD, patients with GAC/HGD were more likely to be older, men, low-income earners, uninfected with *H. pylori*, and have less reported gastritis and more reported polyp histories.

**Table 1:** Patients’ characteristics.

Variables	no-CAG (1)	CAG (2)	IM (3)	LGD (4)	GAC/HGD (5)	*P* (1 vs. 2)	*P* (2 vs. 3)	*P* (3 vs. 4)	*P* (4 vs. 5)
	*n* = 1,739	*n* = 3,409	*n* = 1,757	*n* = 2,239	*n* = 182				
**Covariates**									
Age	50.6 ± 7.4	51.4 ± 7.6	52.3 ± 7.9	52.5 ± 8.1	57.8 ± 7.1	**<0.001**	**<0.001**	0.39	**<0.001**
Male	866 (49.8%)	1794 (52.6%)	935 (53.2%)	1,420 (63.4%)	140 (76.9%)	0.05	0.69	**<0.001**	**<0.001**
Married	1,661 (95.5%)	3,264 (95.7%)	1,662 (94.6%)	2,116 (94.5%)	171 (94.0%)	0.7	0.06	0.9	0.75
Illiteracy	255 (14.7%)	523 (15.3%)	383 (21.8%)	457 (20.4%)	35 (19.2%)	0.52	**<0.001**	0.29	0.7
Farmers	1,508 (86.7%)	3,104 (91.1%)	1,630 (92.8%)	2,108 (94.1%)	177 (97.3%)	**<0.001**	**0.035**	0.08	0.08
Annual family income*	2.0 (1.0, 3.0)	2.0 (1.0, 3.0)	2.0 (1.0, 3.0)	2.0 (1.0, 3.0)	1.5 (0.8, 2.8)	**<0.001**	0.42	0.39	**<0.001**
BMI						0.5	0.6	0.8	**<0.001**
Underweight	39 (2.2%)	64 (1.9%)	36 (2.0%)	54 (2.4%)	13 (7.1%)				
Normal	875 (50.3%)	1,675 (49.1%)	895 (50.9%)	1,153 (51.5%)	98 (53.8%)					
Overweight	669 (38.5%)	1,377 (40.4%)	682 (38.8%)	860 (38.4%)	60 (33.0%)				
Obese	156 (9.0%)	292 (8.6%)	144 (8.2%)	172 (7.7%)	11 (6.0%)				
*Helicobacter pylori* infection	764 (44.2%)	1,849 (54.4%)	1,000 (57.0%)	1,311 (58.7%)	87 (48.1%)	**<0.001**	0.08	0.3	**0.006**
**Life styles**									
Smoking or ever	602 (34.7%)	1,245 (36.6%)	708 (40.4%)	1,061 (47.6%)	96 (52.7%)	0.17	**0.008**	**<0.001**	0.18
Drinking	69 (4.0%)	186 (5.5%)	100 (5.7%)	148 (6.6%)	7 (3.8%)	**0.02**	0.73	0.23	0.14
Eating hot food	972 (55.9%)	2,050 (60.2%)	1,135 (64.6%)	1,362 (60.8%)	110 (60.4%)	**0.003**	**0.002**	**0.015**	0.92
Eating fast	349 (20.1%)	680 (19.9%)	404 (23.0%)	499 (22.3%)	41 (22.5%)	0.92	**0.011**	0.6	0.94
**Self-reported diseases**									
Gastritis	290 (16.7%)	943 (27.7%)	654 (37.2%)	883 (39.4%)	27 (14.8%)	**<0.001**	**<0.001**	0.15	**<0.001**
Peptic ulcer	26 (1.5%)	94 (2.8%)	58 (3.3%)	116 (5.2%)	10 (5.5%)	**0.005**	0.27	**0.004**	0.85
Hepatitis	16 (0.9%)	34 (1.0%)	19 (1.1%)	43 (1.9%)	2 (1.1%)	0.79	0.78	**0.033**	0.43
Pancreatitis	3 (0.2%)	1 (0.0%)	3 (0.2%)	2 (0.1%)	0 (0.0%)	0.08	0.08	0.47	0.69
Gallbladder diseases	206 (11.8%)	385 (11.3%)	250 (14.2%)	316 (14.1%)	22 (12.1%)	0.56	**0.002**	0.92	0.45
Polyp	29 (1.7%)	58 (1.7%)	39 (2.2%)	57 (2.5%)	10 (5.5%)	0.93	0.19	0.5	**0.02**
High blood pressure	271 (15.6%)	536 (15.7%)	298 (17.0%)	383 (17.1%)	24 (13.2%)	0.9	0.25	0.9	0.17
Diabetes	45 (2.6%)	113 (3.3%)	64 (3.6%)	73 (3.3%)	8 (4.4%)	0.15	0.54	0.51	0.41
Anemia	15 (0.9%)	30 (0.9%)	20 (1.1%)	22 (1.0%)	2 (1.1%)	0.95	0.37	0.63	0.88
Family history of gastric cancer	5 (0.3%)	13 (0.4%)	6 (0.3%)	12 (0.5%)	0 (0.0%)	0.59	0.82	0.36	0.32

*Annual family income in ten thousand Yuan. BMI, body mass index; no-CAG, chronic non-atrophic gastritis; CAG, chronic atrophic gastritis; IM, intestinal metaplasia; LGD, low-grade dysplasia; HGD, high-grade dysplasia; GAC, gastric adenocarcinoma. Bold entries represent *p* values that are significant.

### 3.2. Risk factors associated with the development of GAC/HGD

Overall, age, male sex, having farming as an occupation, and a history of polyps were strong risk factors for GAC/HGD with ORs of 2.79–3.41 when compared with the no-CAG group in the adjusted analysis. *H. pylori* infection, smoking, drinking, eating hot food, eating fast, and having a history of peptic ulcer appeared to be risk factors, with a 1.13–2.39-fold odds of developing GAC/HGD, although these associations were not significant. Increased family income had a significant protective effect. For every ten-thousand increase in family income, the OR was reduced by 18% (OR: 0.82, 95% CI: 0.73–0.93; Table 2).

**Table 2:** Effect of varying factors on each progression.

Variables	no-CAG to CAG	CAG to IM	IM to LGD	LGD to GAC/HGD	no-CAG to GAC /HGD
	OR (95% CI)	OR (95% CI)	OR (95% CI)	OR (95% CI)	OR (95% CI)
Age per 10 years	**1.15 (1.06–1.26)**	**1.09 (1.01–1.19)**	0.99 (0.91–1.08)	**2.26 (1.81–2.82)**	**3.07 (2.41–3.91)**
Male	1.06 (0.90–1.25)	0.98 (0.83–1.16)	**1.65 (1.37–1.98)**	1.57 (0.99–2.51)	**2.79 (1.73–4.50)**
Illiteracy	0.98 (0.82–1.18)	**1.50 (1.27–1.78)**	1.12 (0.94–1.34)	0.67 (0.43–1.04)	1.02 (0.62–1.66)
Farmers	**1.35 (1.11–1.64)**	1.12 (0.90–1.41)	**1.38 (1.06–1.79)**	1.63 (0.63–4.20)	**3.37 (1.32–8.64)**
Annual family income*	**0.95 (0.92–0.98)**	0.97 (0.94–1.00)	1.02 (0.98–1.06)	0.88 (0.78–1.00)	**0.82 (0.73–0.93)**
Underweight	0.82 (0.54–1.25)	1.01 (0.66–1.54)	1.20 (0.78–1.87)	**2.01 (1.02–3.96)**	**2.81 (1.26–6.29)**
Overweight	1.10 (0.97–1.25)	0.91 (0.80–1.03)	0.98 (0.86–1.12)	0.98 (0.69–1.40)	0.98 (0.68–1.42)
Obese	0.96 (0.77–1.19)	0.89 (0.71–1.10)	0.91 (0.72–1.16)	0.96 (0.49–1.88)	0.74 (0.37–1.47)
*Helicobacter pylori* infection	**1.51 (1.34–1.70)**	**1.14 (1.01–1.28)**	1.08 (0.95–1.22)	0.81 (0.59–1.12)	1.25 (0.89–1.76)
Smoking or ever	1.02 (0.87–1.20)	**1.26 (1.07–1.49)**	1.00 (0.84–1.19)	0.95 (0.65–1.39)	1.29 (0.87–1.93)
Drinking	**1.46 (1.08–1.96)**	1.06 (0.82–1.38)	1.02 (0.78–1.34)	0.71 (0.32–1.59)	1.13 (0.47–2.72)
Eating hot food	**1.23 (1.09–1.39)**	**1.17 (1.03–1.32)**	**0.86 (0.75–0.98)**	1.06 (0.76–1.47)	1.36 (0.96–1.94)
Eating fast	0.99 (0.85–1.15)	**1.23 (1.07–1.43)**	0.96 (0.82–1.12)	1.12 (0.76–1.66)	1.43 (0.94–2.17)
Gastritis history	**1.91 (1.65–2.22)**	**1.54 (1.36–1.75)**	1.13 (0.99–1.29)	**0.27 (0.18–0.42)**	0.73 (0.45–1.19)
Peptic ulcer history	**1.78 (1.14–2.78)**	1.07 (0.76–1.50)	**1.60 (1.15–2.22)**	0.88 (0.44–1.78)	2.39 (0.99–5.74)
Polyp history	1.05 (0.66–1.66)	1.34 (0.88–2.04)	1.19 (0.78–1.81)	1.88 (0.90–3.92)	**3.41 (1.46–7.99)**
Gallbladder diseases history	0.93 (0.77–1.13)	**1.22 (1.02–1.46)**	1.09 (0.91–1.32)	0.79 (0.48–1.30)	1.01 (0.59–1.72)

*Annual family income in ten thousand Yuan.

BMI, body mass index; no-CAG, chronic non-atrophic gastritis; CAG, chronic atrophic gastritis; IM, intestinal metaplasia; LGD, low-grade dysplasia; HGD, high-grade dysplasia; GAC, gastric adenocarcinoma. Bold entries represent *p* values that are significant.

### 3.3. Risk factors associated with the development of CAG in patients with no-CAG

Age, having farming as an occupation, *H. pylori* infection, alcohol consumption, eating hot food, and history of gastritis and peptic ulcer were risk factors for the development of CAG among non-CAG patients. For every 10-year increase in age, the odds increased by 15% (OR: 1.15, 95% CI: 1.06–1.26). Having farming as an occupation, *H. pylori* infection, drinking, and eating hot food significantly increased the odds by 23 to 51%. Histories of gastritis and peptic ulcers were strong risk factors with 1.91 and 1.78 higher OR (OR: 1.91, 95% CI: 1.65–2.22; OR: 1.78, 95% CI: 1.14–2.78). Increased annual family income was a protective factor. For every ten-thousand increase in annual family income, the OR decreased by 5% (OR: 0.95, 95% CI: 0.92–0.98; Table 2).

### 3.4. Risk factors associated with the development of IM in patients with CAG

Age, illiteracy, *H. pylori* infection, smoking, eating hot food, eating quickly, and having a history of gastritis and gallbladder disease were risk factors for the development of IM among CAG patients. For every 10-year increase in age, the OR increased by 9% (OR: 1.09, 95% CI: 1.01–1.19). Illiteracy, *H. pylori* infection, smoking, eating hot food, eating quickly, as well as a history of gastritis and gallbladder disease, increased the odds by 14–50% (Table 2).

### 3.5. Risk factors associated with the development of LGD in patients with IM

Male sex, but not age, was associated with the development of LGD in IM patients. Males with IM had 1.65 higher odds of developing LGD than females (OR: 1.65, 95% CI: 1.37–1.98). Having farming as an occupation and having a history of peptic ulcers were also associated with the development of LGD in IM patients, with an increased odds of 38% (OR: 1.38, 95% CI: 1.06–1.79) and 60% (OR: 1.60, 95% CI: 1.15–2.22), respectively. The habit of eating hot food did not seem to be associated with the development of LGD in IM patients, although it was slightly significant (OR: 0.86, 95% CI: 0.75–0.98; Table 2).

### 3.6. Risk factors associated with the development of GAC/HGD in patients with LGD

Age was a strong risk factor for the development of GAC/HGD among patients with LGD. For every ten-year increase in age, the odds increased by 126% (OR: 2.26, 95% CI: 1.81–2.82). Male sex appeared to be associated with GAC/HGD. Males were 1.57 times more likely to develop GAC/HGD than females among LGD patients (OR: 1.57, 95% CI: 0.99–2.51). Moreover, having a history of polyps seemed to be a risk factor with an OR of 1.88 (OR: 1.88, 95% CI: 0.90–3.92). Patients with GAC/HGD were more likely to be underweight (OR: 2.01, 95% CI: 1.02–3.96) and less likely to report a history of gastritis (OR: 0.27, 95% CI: 0.18–0·42; Table 2).

## 4. Discussion

**Figure 2:**
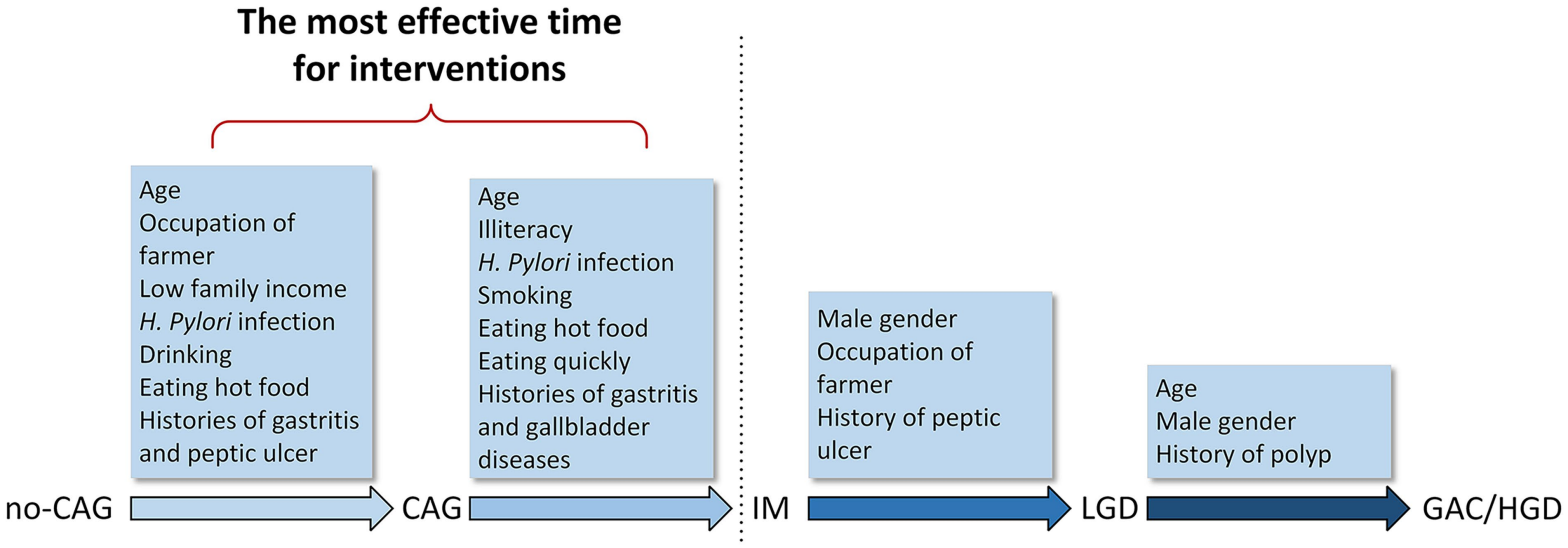
Risk factors in the development of gastric adenocarcinoma. Different groups of risk factors associated with different stages from no-CAG to GAC/HGD. no-CAG, chronic non-atrophic gastritis; CAG, chronic atrophic gastritis; IM, intestinal metaplasia; LGD, low-grade dysplasia; HGD, high-grade dysplasia; GAC, gastric adenocarcinoma.

In the present study, we examined risk factors associated with different disease stages in the development of GAC. We found that different groups of risk factors were associated with separate disease stages (Figure 2). Age, having farming as an occupation, low annual family income, *H. pylori* infection, drinking, eating hot food, and gastritis or peptic ulcer history were associated with the development of CAG. Age, illiteracy, *H. pylori* infection, smoking, eating hot food, eating quickly, and gastritis or gallbladder diseases history were associated with the progression to IM from CAG. Male sex, having farming as an occupation, and a history of peptic ulcers were associated with the development of LGD from IM. Age and male sex appeared to be risk factors associated with the progression to GAC/HGD from LGD.

Studies have shown that GAC is more common in the elderly and in men (Derakhshan et al., 2009; Piazuelo and Correa, 2013; Suh et al., 2017). In concordance with previous studies, our results show that age increases the risk of development from no-CAG to CAG, CAG to IM, and LGD to GAC/HGD. As aging is associated with prior progression, GAC/HGD was more likely to occur than premalignant lesions in the elderly, and the OR between patients with GAC/HGD and no-CAG was approximately twice than that between each of the two consecutive groups for premalignant lesions. Our results also confirm the observations of a male predominance in GAC. Patients with GAC/HGD were 2.79 times more likely to be male, and the predominance began from the progression from IM to LGD, which means that male patients with IM should undergo more intensive endoscopic examinations.

People with low socioeconomic status has been shown to be twice as likely to develop GAC (Ji and Hemminki, 2006; Nagaich, 2018; Liu et al., 2019). We found that illiteracy, having farming as an occupation, and low annual family income was associated with different progressions of the disease. Farmers were 3.37 times more likely to develop GAC/HGD, and the risk of GAC/HGD decreased by 18% for every ten-thousand increase in annual family income in these patients compared with no-CAG patients. The reason why low socioeconomic status increases the risk of GAC is not fully understood, although related characteristics such as poor living conditions, low health awareness, and *H. pylori* infection may be the main causes (Nagel et al., 2007; Lagergren et al., 2016; Lyons et al., 2019).

Studies on the association between GAC and obesity have shown conflicting results. A meta-analysis including 24 prospective studies showed that obesity and being overweight were associated with the development of gastric cardiac cancer, but not with noncardiac cancer (Chen et al., 2013). In our study, because of the small number of gastric cardiac cancers, we combined them. However, we did not find an association between obesity and being overweight with the development of GAC/HGD nor any progression of premalignant lesions.

*Helicobacter pylori* has been classified as a group 1 carcinogen that leads to gastric adenocarcinoma by the WHO. Ever since the identification of *H. pylori* as a causative agent of gastritis, it was recognized that Correa’s cascade is initiated and sustained by *H. pylori* infection (Piazuelo and Correa, 2013). *H. pylori* eradication in patients with CAG and IM has been recommended by the guidelines in Asia and Europe (Yoon and Kim, 2015). Some studies have shown that *H. pylori* eradication can delay or reverse mucosal atrophy and IM, thereby diminishing the progression of IM (Leung et al., 2004; Hwang et al., 2018; Kim, 2019). However, other studies have shown that eradication of *H. pylori* can only regress chronic gastritis and CAG, and that eradication at the stage of IM is less effective, with diseases still more likely to progress (Rokkas et al., 2007; Lee et al., 2013). Our results show that although high infection rates were observed in every disease stage, a significant association was only observed in the development from no-CAG to IM, which verifies the hypothesis that *H. pylori* is not directly associated with gastric carcinogenesis. Rather, *H. pylori* triggers a multistep process, and *H. pylori*-induced no-CAG, CAG, and IM provide the seed of cascade leading to GAC, which would progress continuously even in the absence of *H. pylori* (Park and Kim, 2015). Therefore, *H. pylori* should be eradicated before the IM stage. Practically, the Japanese healthcare system has expanded the application of medical insurance to eradicate *H. pylori* in all patients with chronic gastritis (Kobayashi et al., 2015).

Smoking has been reported to be a risk factor for GAC, causing a 60 and 20% increase in GAC risk for men and women, respectively (Nagaich, 2018). In premalignant lesions, smoking has been reported to be associated with IM, but not with CAG (Kato et al., 2004; Peleteiro et al., 2007; Kim et al., 2019; Muhsen et al., 2019). In our study, we did not find a significant association between CAG and smoking. However, smoking appears to increase the risk for the progression from CAG to IM. In addition, smokers were 1.29 times more likely to develop GAC/HGD when compared with the no-CAG group, although the association was not significant. Therefore, smoking may indirectly cause the development of GAC/HGD by increasing the risk of IM.

Drinking is a risk factor for the development of adverse health conditions. The relationship between drinking and GAC has been inconsistently reported. Some studies have shown that long-term alcohol consumption increases the risk of GAC/HGD (Duell et al., 2011; Fang et al., 2015). However, no significant associations have also been reported in other studies (Tramacere et al., 2012; Wang et al., 2017, 2018). Our results did not find an association between drinking and GAC/HGD when compared with the no-CAG group. However, a positive association between drinking and CAG was observed, which may be explained by the fact that ethanol intake causes mucosal damage and destruction of the glands, leading to a reduction in acid secretion and chronic inflammation, eventually causing the development of CAG (Na and Lee, 2017). Therefore, drinking may play a role in GAC carcinogenesis.

Our results showed that eating hot food was associated with the progression to CAG and IM as well as appeared to increase the risk of GAC/HGD, which means that thermal irritation promotes gastric carcinogenesis in the CAG to IM pathway (La Vecchia et al., 1990). In addition, eating quickly was associated with the development of IM and showed a positive, but not significant, association with GAC/HGD.

Gastritis, gastric ulcers, and gastric polyps are precancerous diseases of gastric cancer. Having a history of gastritis was a strong risk factor for GAC because chronic superficial gastritis and CAG are the first two diseases of the cascade of precursor lesions of GAC. Our study showed that a history of gastritis was associated with CAG and IM, and that it was likely to be associated with LGD, although this association was not significant. Due to the lack of awareness regarding the necessity to undergo a regular endoscopic examination as well as having a low family income, patients with GAC/HGD in Northwest China seldom underwent endoscopic examinations before they were diagnosed with GAC/HGD; thus, an inverse association between having a history of gastritis and the development of GAC/HGD was observed when compared with LGD patients, and an insignificant association between history of gastritis and GAC/HGD was also observed when compared with no-CAG. Approximately 5% of gastric ulcers develop GAC (Committee ASoP et al., 2010). It is generally believed that the mucosal epithelium at the edge of the ulcer can be cancerous due to repeated destruction and repair and stimulation from tumorigenic factors. Our results showed that patients with a history of peptic ulcers were 3.13 times more likely to develop GAC/HGD, which may be a consequence of a history of peptic ulcers promoting the development of CAG and LGD. About 6–47% of gastric polyps will develop GAC. All gastric polyps, except inflammatory polyps, may develop GAC/HGD, especially adenomatous and hyperplastic polyps. Studies have shown that adenomatous polyps are more likely to develop GAC, are often accompanied by IM or dysplasia, and may even coexist with GAC (Cheesman et al., 2017). Our results show that patients with a history of polyp had 4.20 times higher odds of developing GAC/HGD. Gallbladder disease plays a role in the gastric cancer pathway (Kang et al., 2017). Our study indicates that gallbladder diseases are associated with the risk of IM.

IM appears to be a special stage of gastric carcinogenesis. Many factors, such as *H. pylori* infection, low family income, smoking, drinking, eating hot food, and eating quickly were significantly associated with the progression from no-CAG to IM, which then became insignificant after the stage of IM. Therefore, the prevention and treatment of no-CAG and CAG is pivotal for decreasing the incidence of GAC. The eradication of *H. pylori* and control of other risk factors must be conducted for patients with no-CAG and CAG. It would be more effective to treat *H. pylori* infection and eliminate other risk factors in younger people before no-CAG develops (Figure 2).

Our study has several strengths. First, we included patients from the Wuwei Cohort, which is a population-based study. Therefore, the samples in our study were representative of patients in the general population. Second, the relatively large sample size of this study permitted us to detect relatively smaller associations of risk factors with GAC/HGD. This study has some limitations. First, our results should be regarded as hypothesis generation. We used cross-sectional data to examine the effect of different risk factors on the progression of disease stages by comparing two consecutive disease stages. Therefore, the results indicate an epidemiological risk association rather than a time-dependent progression among different disease stages. We hope our study will encourage the conduct of additional studies on the effect of different risk factors on the progression from no-CAG to GAC in the general population. Second, we found that age, male sex, and polyp history appeared to be risk factors associated with the development from IM to LGD, which may not be comprehensive. Premalignant lesions accounted for 78.9% of the total pathological diagnoses in the Wuwei Cohort, while CAG, IM, and LGD accounted for 45.4, 23.4, and 30.1% of premalignant lesions, respectively, indicating that a large number of premalignant lesions are the basis for the high incidence of GAC. In addition, patients with IM could progress to LGD on a large scale. Hence, genetic and environmental factors may play important roles in this progression besides age, male sex, and polyp history, although this requires further investigation.

## 5. Conclusion

In conclusion, our results show that different groups of risk factors appear to be associated with separate stages from no-CAG to GAC/HGD. Most risk factors seem to function more as a set of switches that initiate GAC carcinogenesis, and the most effective time point for interventions is before the development of IM. Nevertheless, risk factors related to the progression from IM to LGD still require further study.

## Data availability statement

The raw data supporting the conclusions of this article will be made available by the authors, without undue reservation.

## Ethics statement

The studies involving human participants were reviewed and approved by the Ethics Committee of The First Hospital of Lanzhou University. The patients/participants provided their written informed consent to participate in this study.

## Author contributions

ZC, YaZ, PF, MLi, WLiu, and HY conceptualized and wrote the paper. YaZ and JL analyzed and interpreted the data. XinL, ZZ, ZW, YW, RJ, QiG, YY, JiZ, XiaL, FA, LL, YL, XW, JuZ, QuG, QL, ML, QR, XH, HL, HZ, YuZ, XG, XS, JW, ZH, and SX acquired the data and provided technical, or material support. XH, JL, and YoZ supervised the cohort and the study. YoZ obtained funding. All authors provided critical feedback and edits to subsequent revisions, and approved the final draft of the manuscript.

## Funding

This work was supported by the Ministry of Science and Technology of the People’s Republic of China (2012GS620101).

## Conflict of interest

The authors declare that the research was conducted in the absence of any commercial or financial relationships that could be construed as a potential conflict of interest.

## Publisher’s note

All claims expressed in this article are solely those of the authors and do not necessarily represent those of their affiliated organizations, or those of the publisher, the editors and the reviewers. Any product that may be evaluated in this article, or claim that may be made by its manufacturer, is not guaranteed or endorsed by the publisher.

## Abbreviations

BMI: body mass index;
CAG: chronic atrophic gastritis;
GAC: gastric adenocarcinoma;
HGD: high-grade dysplasia;
IM: intestinal metaplasia;
LGD: low-grade dysplasia;
no-CAG: chronic non-atrophic gastritis.
